# Pest Status and Distribution of the Stem Borer, *Dectes texanus*, in Kansas

**DOI:** 10.1673/031.010.19801

**Published:** 2010-11-15

**Authors:** Lawrent L. Buschman, Phillip E. Sloderbeck

**Affiliations:** Department of Entomology, Kansas State University and Kansas State University Southwest Research-Extension Center

**Keywords:** geographic, soybean stem borer, sunflower stem borer, sunflower stem girdler

## Abstract

The Dectes stem borer, *Dectes texanus* LeConte (Coleoptera: Cerambycidae), is currently receiving increased attention as a pest of soybeans in the Great Plains of North America. Field surveys were conducted in 1999 and in 2008 to record the distribution of this pest in Kansas. These surveys documented an increase in the abundance of the pest and an expansion in the range of this insect westward and eastward. The percentage of fields with more than 50% of plants infested also increased from 4% in 1999 to 11% in 2008. The far eastern counties still had surprisingly few infested fields even though much of the Kansas soybean acreage is located in these counties. It is not clear if *D. texanus* simply haven't expanded into eastern Kansas yet or if there is an ecological barrier that keeps them from doing so. Field crop entomologists from across eastern North America were sent an email questionnaire and their responses indicate that this pest is now well established as a pest of soybeans in at least 14 states across eastern North America.

## Introduction

The Dectes stem borer, *Dectes texanus* LeConte (Coleoptera: Cerambycidae), is native to most of eastern North America, where it has been recorded on soybeans (*Glycine max* L.), sunflowers (*Helianthus* spp.), cocklebur (*Xanthium* spp.), and ragweed (*Ambrosia* spp.) (Hatchet et al. 1975; Campbell 1980; Lentz 1994). The larval stages of *D. texanus* tunnel and feed on the pith inside the plant petioles and stems from late July to mid October (Hatchet et al. 1975). At the end of the season, as the plant matures and dries out, *D. texanus* tunnel to the base of the plant and cut the lower stem from inside. This act is known as girdling and causes the plant to fall to the ground. Mature larvae spend the winter in the plant bases below the soil line. In summer (May and June) the larvae pupate and the adult long-horned beetles emerge in late June and July and fly to new host plants. The beetles feed on plant epidermis of newly expanded tissue before they lay their eggs in the plant. The eggs are inserted all the way into the pith of the leaf petiole. The eggs hatch in a few days, and the cycle starts over. There is a single generation each year. The basic biology of this beetle has been well described (Patrick 1973; Hatchet et al. 1975; Richardson 1975; Campbell and Van Duyn 1977; Rogers 1985; Michaud and Grant 2005).

*D. texanus* has become a pest of soybeans and cultivated sunflowers in North America (Hatchet et al. 1975; Rogers 1985). This insect causes most of its damage when the larvae girdle the stem, causing the plant to fall to the ground or ““lodge””. This makes harvest more difficult and leads to significant harvest losses. Fortunately, growers who are aware that their fields are infested can harvest the field promptly and thus avoid significant lodging losses. Some research suggests a 10 to 15% yield reduction associated with the tunneling activity (Daugherty and Jackson 1969; Buschman et al. 2006; Davis et al. 2008a). However, the overall yield loss from tunneling and lodging associated with this pest is not well documented. There is a concern because management options for this pest are limited (Lentz 1994; Sloderbeck et al. 2003; 2008). Older recommendations suggest using crop rotation and stubble destruction to reduce damage from this pest, but these cultural controls are not compatible with current farming practices. For example, rotation may be useful when soybean acreage is limited and soybean fields are isolated, but it appears to lose effectiveness when the regional acreage increases to the extent that *D. texanus* can easily find soybean fields each year. They are reasonably strong flyers and can infest other soybean fields within several miles, but they are not known to undergo long distance dispersal. Stubble destruction is no longer an acceptable practice because the stubble is needed for soil conservation and compliance with legal requirements for minimum residue coverage. Early insecticide efficacy trials showed that few insecticides control this pest (Campbell and Van Duyn 1977), probably because beetles re-infest treated small plots so quickly. Recently, Sloderbeck et al. (2004) demonstrated that two applications of a pyrethroid insecticide like lambda-cyhalothrin (Warrior™™) applied to large plots or whole fields could reduce *D. texanus* infestations up to 80%. We also were able to demonstrate that a systemic insecticide could be used to manage the pest, but this insecticide has not yet received registration for use on commercial soybeans (Buschman et al. 2005; Davis et al. 2008b; Niide et al. 2008). To date, there are no High Plains- or Midwestern-adapted soybean varieties that exhibit host plant resistance to *D. texanus* (Sloderbeck et al. 2003; Kaczmarek 2003), but there are claims of host plant resistance in soybeans adapted to the southeast (Richardson 1975).

Occurrence of *D. texanus* is not well documented. Campbell (1980) reported major infestations along the Mississippi river valley and on the East Coast, and Richardson (1975) documented infested counties in North Carolina. It is interesting to note that Campbell (1980) did not include Kansas in his map of regions with damaging infestations of *D. texanus* in soybeans. Records of this pest have been accumulating in Kansas over the past two decades. This insect was first documented as a soybean pest in Kansas in early October 1985, when serious lodging (38%) was found in a soybean field in Edwards County and later in four other counties (Barton, Kiowa, Ford, and Pawnee) (Bell 1985a, 1985b). By 1991 the pest had been detected in 16 counties from southwest Kansas through south central and north central Kansas (Barton, Edwards, Finney, Ford, Gray, Harvey, Kiowa, McPherson, Meade, Mitchell, Pawnee, Pratt, Saline, Sedgwick, Stafford, and Stanton) (Bell 1991a, 1991b). From 1991 to 1998, only three more counties (Ellsworth (Bell 1994), Republic (Randy Higgins, personal communication 1998), and Rice (anonymous observation from author's records)) were added to the list, to bring the total number of infested counties to 19. By 2002, the pest had been recorded in at least 41 counties (Sloderbeck et al. 2003).

In recent years, reports and site visits have confirmed that fields in many Kansas counties now routinely experience heavy damage from *D. texanus* with infestations ranging from 50 to 100% of plants. Dramatic lodging can occur if harvest is delayed by rainy weather. The distribution of these damaging populations appears to be expanding in Kansas. *D. texanus* is also recognized as a pest in sunflowers (Michaud et al. 2007).

For some unknown reason, *D. texanus* populations have remained low in eastern Kansas, except for one early unconfirmed report in southeast Kansas (Bell 1986). This situation is surprising because the soybean acreage is extensive in that region and *D. texanus* has long been recognized as a pest in soybeans further to the east in the boot heel of Missouri (Hatchet et al. 1975). *D. texanus* are cryptic (spending most of their time tunneling inside the plant) so they may be present without soybean growers (or entomologists) being aware of their presence.

The purpose of this study was to document the pest status of *D. texanus* in Kansas by conducting field surveys of infestations. These surveys also sought to determine whether *D. texanus* were really absent from eastern Kansas or simply unreported from this region. In addition an e-mail questionnaire was sent to entomologists across eastern North America to determine the current pest status of this insect.

## Methods and Materials

In 1999, and again in 2008, a field survey for *D. texanus* was conducted by visiting soybean fields across Kansas. The objective was to visit two soybean fields in 1999 and two to four fields in 2008 in each county. However, it was difficult to find even one soybean field in some counties. There were many counties in which only one field was available to sample. One or two persons would enter each field 10–15 m and each person inspected 10 or 20 plants for a total of 10 to 20 plants in each field. The plants were visually inspected or split to look for *D. texanus* or their tunneling. The sample size (10 to 20 plants per field) may seem rather small, but the authors have found that it is sufficient to determine the infestation in that part of the field (personal observation). However, there can be large differences across a field so one would need to visit the different parts of the field to get an overall field estimate. That was not practical in this survey because access to only one part of the field was usually available. Since we were not as much concerned with the precision of the estimate for the individual fields, but with county-wide levels of infestation, we chose to increase the number of fields visited in 2008 and to increase the county sample size, especially for counties with larger soybean acreages. In 2008 up to 4 fields per county were visited, this was especially the case in the eastern counties which had large soybean acreages.

Reports of *D. texanus* from county agents, crop consultants, and chemical company representatives were collected between 1999 and 2007. Additionally, fields were visited in counties where *D. texanus* had not been previously recorded to determine whether they were present.

To document the distribution of *D. texanus* across North America, a questionnaire was sent by e-mail to field crop entomologists in states east of the Rocky Mountains. Respondents were asked to identify the pest status of *D. texanus* in their state and to answer several questions about the management of the pest. Responses were not received from a few states so infestation information from published notes in these states was used. The questionnaire focused on *D. texanus* occurrence in soybeans, but there were also some notes on its occurrence in sunflowers.

The two Kansas field surveys were analyzed statistically using a one-way ANOVA across the two sample years using a mixed model. Data for each year were also analyzed by crop reporting district in a one-way ANOVA; districts that had no *D. texanus* were excluded from analysis. Data were transformed using the log (x + 1) transformation and analyzed using the MSTAT Statistical Program (MSTAT Development Team 1988).

## Results

In 1999, 74 fields from 59 of 105 counties in Kansas were visited. Infestations of *D. texanus* were found in 10 counties (Table 1). The highest percentage of fields infested was in the south central crop reporting district, where 4 out of 7 fields were infested. This district also had the highest percentage of plants infested, averaging 15.7%. A total of 1480 plants were evaluated and 76 plants were infested. *D. texanus* were not detected in four crop reporting districts: northwest, northeast, east central, and southeast. In the counties reported to have been infested prior to this survey, only 7 of 14 fields were infested with *D. texanus* in this sample. Stem borers were detected in three previously uninfested counties: Cloud, Hodgeman, and Ness.

Between 1999 and 2008, 30 counties were added to the known distribution of *D. texanus* in Kansas (Barber, Clark, Clay, Decatur, Dickinson, Ellis, Franklin, Geary, Gove, Graham, Grant, Gray, Haskell, Jewell, Kearny, Lincoln, Morris, Norton, Ottawa, Rawlins, Reno, Scott, Seward, Rush, Russell, Sheridan, Sherman, Stevens, Thomas, Trego, and Washington). Thus, by 2005, *D. texanus* had been reported from 52 of 105 counties in Kansas.

In 2008, 220 fields from 92 of 105 counties in Kansas were visited. *D. texanus* were found in 87 fields in 47 counties (Table 1). The highest levels of infestations were in the southwest reporting district, which had the highest percentage of infested fields (82%) and the highest percentage of infested plants (40.3%). However, the north central district was also heavily infested, with 78% of fields infested and 35.1% of plants infested. In total, 2325 plants were inspected and 360 were infested. *D. texanus* larvae were detected in all crop reporting districts, except for the southeast, but even there one adult beetle was observed during the survey. *D. texanus* infestation in the three eastern crop reporting districts remained very low; only 0.8% of the 106 fields in these three districts were infested. Of the 52 counties previously reported to be infested, 44 were sampled and *D. texanus* were detected in 36 (81%). This survey also documented the presence of *D. texanus* in 12 new counties (Comanche, Greenwood, Lane, Logan, Osage, Osborne, Riley, Smith, Stevens, Sumner, Wabaunsee, and Wichita).

**Table 1.**
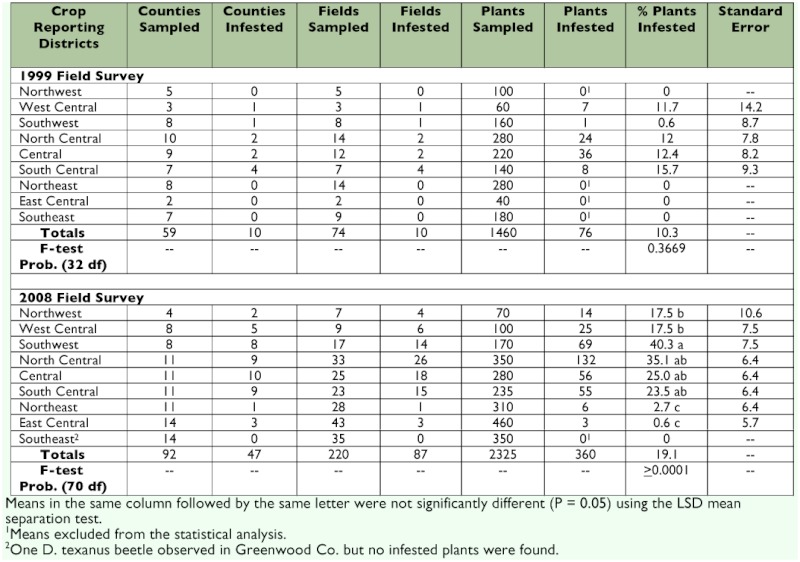
Analysis of field survey data for *Dectes texanus* from Kansas by crop reporting districts.

**Figure 1.**
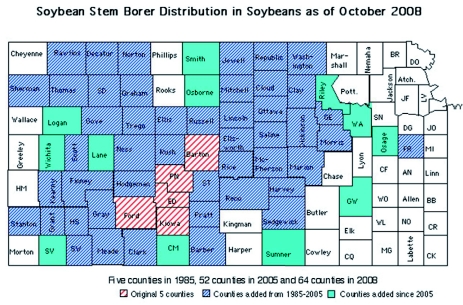
Distribution of *Dectes texanus* in Kansas soybeans as of October 2008. Red diagonally striped counties were reported to be infested in 1985, diagonally striped blue counties were added between 1985 and 1998, diagonally striped counties were added between 1999 and 2005, and solid green counties were added from the 2008 survey. High quality figures are available online.

The known distribution of *D. texanus* on soybeans in Kansas is presented in Figure 1. From the initial infestations detected in five counties by the Kansas State Board of Agriculture in 1985 (red counties), infestation has increased or spread to 64 Kansas counties (diagonally striped blue and solid green counties).

**Figure 2.**
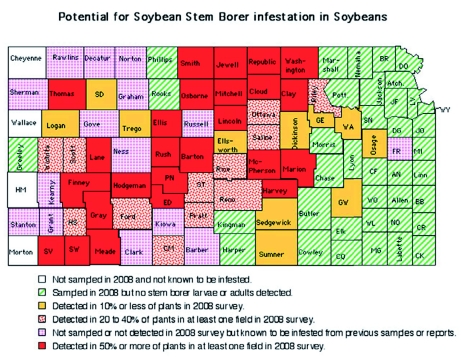
Severity of *Dectes texanus* infestations as observed in the 2008 survey. Solid red counties had high infestations (50% or more plants infested); red stippled counties had significant infestations (20 to 40% of plants infested); counties with horizontal stripes had low levels of *D. texanus* (less that 20%); pink cross hatched counties with vertical stripes were either not sampled or were not found to be infested in the 2008 survey but are known to be infested from previous observations; green diagonally striped counties had no stem borers detected in the in the 2008 survey (and there is no history of infestations); and white counties were not sampled in 2008 and have no history of *D. texanus* infestation. High quality figures are available online.

The severity of *D. texanus* infestations in Kansas by county is presented in Figure 2. The highest infestations occurred across a diagonal region stretching from southwestern to north central Kansas. Counties with significant infestations were in the regions adjacent to the highest infestations. *D. texanus* infestations remained surprisingly low in eastern Kansas, even though the soybean acreage is more extensive in those three crop reporting districts than in most infested counties.

Statistical analysis of the field survey data verified that *D. texanus* infestations were significantly higher in 2008 than in 1999 (P = 0.0001) based on percentage of plants infested. In 1999 no *D. texanus* were detected in four crop reporting districts, but in 2008, the borers were not detected in only one district (Table 1). Excluding uninfested districts, there were no significant differences in the percentage of infested plants across the five districts in 1999 (0.6 to 15.7%; P = 0.3669), but in 2008 infestations were significantly different across the eight districts (0.6 to 40.3%; P > 0.0001). The southwest and north central districts had the highest infestations.

## Discussion

These data indicate that *D. texanus* populations are increasing or expanding across Kansas. This matches the experience of field entomologists, growers, and consultants (Bell 1992). The authors have visited with producers who quit growing soybeans because they could not manage *D. texanus*. This problem has become so extensive that seed companies have recognized a loss of sales and are asking for help in managing this pest. The importance of this pest in Kansas appears to be increasing as Lentz (1994) predicted. Counties with more than 50% of plants infested could expect to have a high potential for lodging in fields where harvest is delayed. Counties with 20 to 50% of plants infested could expect to have potential for significant lodging if harvest is delayed. Counties with detectable infestations could expect to have a low potential for noticeable lodging in isolated fields. However, these surveys included only a few fields in each county, so these estimates are not reliable estimates for pest management in individual fields. Individual fields may have much heavier infestations than those included in the survey, but these data demonstrate that *D. texanus* infestations appear to be increasing and may become more serious in the future.

Although *D. texanus* populations are apparently increasing, the cause is not clearly identifiable. This appears to be a fairly new phenomenon, probably associated with changes in cropping practices. There has been a large increase in soybean acreage in Kansas, from 1.5 million acres in 1985 to 3.3 million acres in 2008 (USDA——NASS Quick Stats 2008). This would increase the likelihood that *D. texanus* would find another soybean field to infest each year. It also increases the likelihood that infested fields would be detected. There has also been an increase in the adoption of no-till farming practices across Kansas, from 2% in 1989 to 21% in 2004 (CTIC 1989 – 2004). Tillage has been shown to reduce overwintering survival of this pest (Campbell and Van Duyn 1977). Thus, the increase in adoption of no-till would tend to increase *D. texanus* population levels. It is interesting to note that the first counties from which *D. texanus* were reported in Kansas were also early adopters of reduced tillage practices to avoid soil erosion on their very sandy soils. Insect population changes can also be associated with changes in annual weather conditions like rainfall and winter temperatures. The increase in *D. texanus* populations may also be evidence of continuing adaptation by this pest to soybeans as a host (Michaud and Grant 2007), since historically it has been primarily associated with composite hosts.

There may also be an increased awareness of the pest because IPM specialists have discussed its presence more frequently in recent years. Larvae of this insect are cryptic (tunneling inside the plant) so soybean growers may not be aware of their presence until they observe plants lodging. When this happens, there is renewed interest and concern about this ““new”” pest.

**Figure 3.**
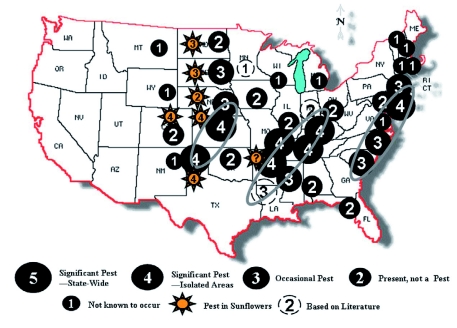
Distribution of *Dectes texanus* in soybeans and sunflowers as reported by soybean entomologists in the 2007 survey. High quality figures are available online.

Responses to the e-mail questionnaire were received from field crop entomologists in 29 states out of the 40 solicited. Additional published information was obtained for another three states. *D. texanus* appear to reach pest status in three zones: 1) Texas panhandle, Kansas, and into Nebraska; 2) along the Mississippi and Ohio Rivers; and 3) along the Atlantic Coast (Figure 3). *D. texanus* do not appear to reach pest status in two zones: 1) the main corn/soybean belt stretching from eastern Kansas to Iowa and Ohio where soybeans are planted extensively, and 2) the southern Gulf Coast from Mississippi to Georgia and Florida. There is also a belt of lower infestations along the Appalachian Mountains. The reason for the low *D. texanus* infestations in the Midwest corn/soybean belt is not known, but could be related to winter kill from low winter temperatures and moisture. However, they are known to occur in this region on wild hosts like ragweed. Perhaps they prefer the wild hosts in these regions and so do not infest soybeans fields as much. If this is true the hypothesized soybean adapted *D. texanus* could disperse into this region and become increasingly important (Michaud and Grant 2007). On the other hand, the increasingly effective weed management in these regions could end up suppressing *D. texanus* populations. There could also be some kind of asynchrony between their life cycle and soybean development in these regions. The low infestations in the southern Gulf Coast are probably the result of insecticide treatments used to control other insect pests in this region. The low infestations in the Appalachians are likely associated with low acreage of soybeans in that area.

E-mail questionnaire respondents reported *D. texanus* as a pest of sunflowers in seven states: North Dakota, South Dakota, Nebraska, Kansas, Colorado, Arkansas, and Texas (Figure 3). This may be more important in sunflowers than in soybeans because sunflower plants lodge more readily and lodged sunflowers are impossible to pick up with current harvesting equipment. However, Michaud et al. (2007) points out that sunflower plants can be large enough to escape complete girdling by *D. texanus* larvae. They also report that they could find no physiological yield loss associated with *D. texanus* feeding in sunflowers —— only lodging losses.

The presence of domestic sunflowers may influence *D. texanus* infestations in soybean. It has been observed that sometimes *D. texanus* can be found in sunflowers but are almost absent from nearby soybean. Michaud et al. (2007) reported that *D. texanus* prefers sunflowers over soybeans to such an extent that they suggest that sunflowers could be used as a trap crop to draw *D. texanus* away from soybeans. This could reduce the soybean infestation in regions with considerable sunflower planting. However, in Kansas the impact of cultivated sunflowers would be limited since significant acreages of sunflowers are grown in only ten counties in Kansas: two in the northwest, two in the southwest, five in the north central, and one in the central crop reporting districts and none in the eastern part of Kansas (USDA-NASS 2008).

There has been some disagreement about what common name to use for *D. texanus*, particularly in extension publications. The survey respondents reported the following usage: ““*D. texanus*”” (17 states), ““Soybean Stem Borer”” (8 states), ““Sunflower Stem Borer”” (2 states), and ““Sunflower Stem Girdler”” (1 state). The problem with including the host in the common name is that the insect attacks multiple hosts, so multiple common names would be needed for the single insect pest. The ““Handbook of Soybean Insect Pests,”” published by the Entomological Society of America (Lentz 1994) uses the common name ““Dectes stem borer””, but the name has not yet been recognized by the ESA common name committee.

The questionnaire respondents reported that the recommendations for monitoring *D. texanus* in their states included: timely harvest (8 states), crop rotation (6 states), nothing known (4 states), variety selection (2 states), foliar sprays (2 states); and tillage, narrow rows, late maturing varieties (1 state each).

It is clear that *D. texanus* is considered a pest in a number of states. Entomologists in these regions are encouraged to conduct field surveys to determine the extent of *D. texanus* infestation in their regions. Entomologists in Missouri, Arkansas, Tennessee, and Kentucky are also reporting the current status of this pest in their states (Tindall et al. 2010). This information will be important in encouraging research into new management options including resistant varieties and insecticides.

## References

[bibr01] Bell KO (1985a). *KS Cooperative Econonomic Insect Survey Report*.

[bibr02] Bell KO (1985b). *KS Cooperative Econonomic Insect Survey Report*.

[bibr03] Bell KO (1986). *KS Cooperative Economic Insect Survey Report*.

[bibr04] Bell KO (1991a). *Cooperative Economic Insect Survey Report*..

[bibr05] Bell KO (1991b). *Cooperative Economic Insect Survey Report*..

[bibr06] Bell KO (1992). *Cooperative Economic Insect Survey Report*..

[bibr07] Bell KO (1994). *Cooperative Economic Insect Survey Report*.

[bibr08] Buschman L, Witt M, Sloderbeck P (2005). Efficacy of in-season applications of systemic insecticide to control Dectes stem borers in soybean.. *Field Day 2005*.

[bibr09] Buschman L, Davis H, Sloderbeck P (2006). Efficacy of in-season applications of systemic insecticide to control Dectes stem borers in soybean.. *Field Day 2005*.

[bibr10] Davis H, Buschman L, Sloderbeck P, Joshi A (2008a). Efficacy of Fipronil Applied as foliar and seed treatment to control Dectes stem borers in soybean, Garden City, KS 2007–South Circle. A.. *Field Day 2005*.

[bibr11] Davis H, Buschman L, Niide T, Joshi A, Khajuria C (2008b). Efficacy of Fipronil Applied as foliar and seed treatment to control Dectes stem borers in soybean, Garden City, KS 2007——Ramsey Field.. *Field Day 2005*.

[bibr12] Campbell WV, Kogan M, Herzog DC (1980). Sampling Coleopterous stem borers in soybean.. *Sampling Methods in Soybean Entomology*..

[bibr13] Campbell WV, Van Duyn JW (1977). Cultural and chemical control of *Dectes texanus texanus* on soybeans.. *Journal of Economic Entomology*.

[bibr14] Conservation Technology Information Center (CTIC). (2004). National crop residue management survey.. http://www.ctic.purdue.edu/.

[bibr15] Daugherty DM, Jackson RD (1969). Economic damage to soybeans caused by a cerambycid beetle.. *Proceedings North Central Branch Entomology Society America*.

[bibr16] Hatchett JH, Daugherty DN, Robbins JC, Barry RM, Houser EC (1975). Biology in Missouri of *Dectes texanus*, A new pest of soybean.. *Annuals of Entomological Society of America*.

[bibr17] Kaczmarek M (2003). The Soybean Stem Borer..

[bibr18] Lentz GL, Higley LG, Boethel DJ (1994). Dectes Stem Borer.. *Handbook of Soybean Insect Pests*.

[bibr19] MSTAT Development Team. (1988). MSTAT-C: A Microcomputer Program for the Design, Management and Analysis of Agronomic Research Experiments..

[bibr20] Michaud JP, Grant AK (2005). The biology and behavior of the long horned beetle, *Dectes texanus*, on sunflower and soybean.. *Journal of Insect Science*.

[bibr21] Michaud JP, Grant AK, Jyoti JL (2007). Impact of the stem borer, *Dectes texanus*, on yield on the cultivated sunflower, *Helianthus annuus*.. *Journal Insect Science*.

[bibr22] Michaud JP, Qureshi JS, Grant AK (2007). Sunflowers as a trap crop for reducing soybean losses to the stalk borer *Dectes texanus* (Coleoptera: Cerambycidae).. *Pest Management Science*..

[bibr23] Niide T, Buschman L, Gordon B, Sloderbeck P, Davis H, Khajuria C (2008). Efficacy of Fipronil Applied as foliar and seed treatment to control *Dectes* stem borers in soybean, Scandia, KS 2007.. *Southwest Research-Extension Center Report of Progress*.

[bibr24] Patrick CR (1973). Observations on the biology of *Dectes texanus texanus* (Coleoptera: Cerambycidae) in Tennessee.. *Journal Georgia Entomological Society*.

[bibr25] Richardson LG (1975). Resistance of Soybeans to a stem borer *Dectes texanus texanus* LeConte.. *MS thesis, Department of Entomology*.

[bibr26] Rogers CE (1985). Cultural Management of *Dectes texanus* (Coleoptera: Cerambycidae) in Sunflower.. *Journal Economic Entomology*.

[bibr27] Sloderbeck P, Buschman L, Higgins R (2004). Soybean stem borer management trials 2001–2003.. *Southwest Research-Extension Center Field Day Report of progress*.

[bibr28] Sloderbeck PE, Kaczmarek M, Buschman LL, Higgins RA, Schapaugh WT, Witt M, Jardine DJ (2003). The Soybean Stem Borer..

[bibr29] Sloderbeck PE, Whitworth J, Michaud JP (2008). Soybean Stem Borer.. *Soybean Insect Management 2008*..

[bibr30] Tindall KV, Stewart S, Musser F, Lorenz G, Bailey W, House J, Henry R, Hastings D, Wallace M, Fothergill K (2010). Distribution of *Dectes texanus* LeConte (Coleoptera: Cerambycidae) in soybeans of Missouri, Western Tennessee, Mississippi, and Arkansas, USA.. *Journal Insect Science*.

[bibr31] USDA -NASS Quick Stats. (2008). http://www.nass.usda.gov/index.asp.

[bibr32] USDA-NASS. (2008). http://www.nass.usda.gov/Charts_and_Maps/Crops_County/pdf/SF-HA08-RGBChor.pdf.

